# Is the Testing System No Longer Effective for Coronavirus Disease 2019? Elucidating the Policy Change in the United Kingdom

**DOI:** 10.31662/jmaj.2022-0143

**Published:** 2022-12-19

**Authors:** Yudai Kaneda, Tetsuya Tanimoto, Kenzo Takahashi, Shiori Akashima, Akihiko Ozaki

**Affiliations:** 1 School of Medicine, Hokkaido University, Hokkaido, Japan; 2 Medical Governance Research Institute, Tokyo, Japan; 3 Teikyo University Graduate School of Public Health, Tokyo, Japan; 4 Faculty of Medical Sciences, Kyushu University, Fukuoka, Japan; 5 Department of Breast Surgery, Jyoban Hospital of Tokiwa Foundation, Fukushima, Japan

**Keywords:** COVID-19, Vaccination, Test frequency, Case fatality rate, United Kingdom

## Abstract

Various studies have reported the vaccine’s efficacy against coronavirus disease 2019; however, there has been little discussion regarding the test frequency since the emergence of the Omicron strain. In this context, the United Kingdom has abolished its free testing program. Our analysis revealed that the decrease in case fatality rate was heavily influenced by vaccination coverage rather than the testing frequency. However, the effectiveness of testing frequency should not be underestimated and therefore needs further validation.

Vaccination and frequent testing and tracking are effective measures to reduce the fatality rate from coronavirus disease 2019 (COVID-19) ^(1), (2)^. Moreover, several studies have reported on the efficacy of the vaccination with the emergence of new variants and with each progressive third and fourth dose administrated. However, the discussions regarding the testing frequency have been limited. Moreover, the government of the United Kingdom (UK) has considered withdrawing the free testing system from April 1, 2022, and will recommend annual vaccination in the future ^(3)^; however, the scientific evidence underlying this decision seems weak. Therefore, we assessed the impact of vaccination coverage and testing frequency on COVID-19 case fatality rates (CFR) using data from nine countries (UK, Croatia, Switzerland, Norway, Sweden, Czechia, Ireland, Denmark, and Lithuania) ^(4)^. Since its adoption, Covid-Pass has been reported to have contributed to approximately 30% reduction in cases and deaths ^(5)^. Therefore, we selected geographically proximate European countries without this system as of February 11, 2022, when the Omicron variant was prevalent, to reduce possible confounders ^(4)^.

Table 1 shows each country’s CFR, vaccine coverage, and test frequency. Fully vaccinated (two doses of vaccination regardless of the type of products) coverage as of February 11, 2022, was high in Denmark (81%) and Ireland (79%), and although it was the lowest Croatia, the vaccination rate was still 54%. Daily number of tests on an average from February 5 to 11, 2022, was highest in Denmark (26.2/1000 individuals) and UK (15.8/1000 individuals), and lowest in Sweden (2.9/1000 individuals) and Croatia (3.0/1000 individuals). CFR was the highest in Croatia (0.878%), while Norway (0.033%) was the lowest during February 5 to 11, 2022. In univariate analysis, there was a strong negative correlation between vaccination coverage and CFR (|r| > 0.75; 95% CI −0.042 to −0.005; p < 0.05; Figure 1). However, frequency of testing was found no statistically significant influence on CFR (|r| < 0.39; 95% CI −0.041 to 0.015; p > 0.1).

**Table 1.** Case Fatality Rate, Vaccine Coverage, and Test Frequency of Each Country.

Country	Case fatality rate (%)	Vaccine coverage (%)	Tests per day (/1,000 population)
United Kingdom	0.257	71.43	15.83
Croatia	0.878	54.35	3
Switzerland	0.088	68.32	7.89
Norway	0.033	73.4	5.34
Sweden	0.293	74.26	2.9
Czechia	0.192	63.61	9.83
Ireland	0.171	79.02	3.45
Denmark	0.054	81.46	26.22
Lithuania	0.156	69.4	8.78

**Figure 1.**
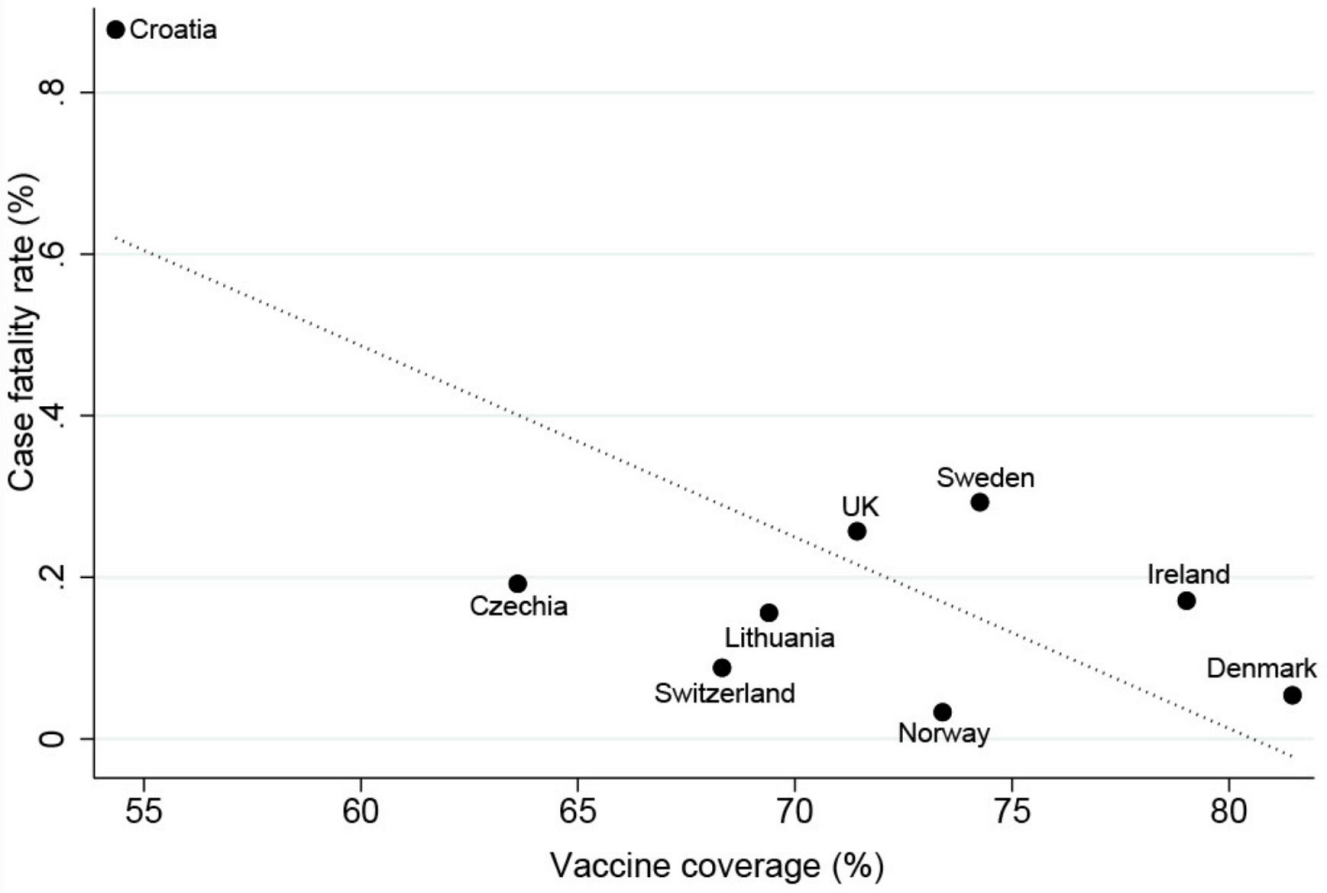
Relation between vaccine coverage and case fatality rate.

Our analyses suggest that vaccination contributes more toward the reduction of CFR than the testing frequency, which partially support the UK’s new policy. This finding suggests that, in terms of CFR after increased vaccination population coverage, the previous consensus that frequent testing is an effective infection control measure is no longer applicable for Omicron variants, which have a shorter incubation period and a lower severity rate compared to the Delta variant. This is an ecological study, and it is difficult to make a definitive statement regarding the causal relationship. Recently, testing is charged in the UK, France, Germany, and elsewhere. However, considering various infection control measures and those individuals that choose not to get vaccinated, the impact of test frequency should be streamlined in the future using local individual data.

## Article Information

### Conflicts of Interest

Akihiko Ozaki received personal fees from MNES Inc. outside the submitted work. Furthermore, Tetsuya Tanimoto received personal fees from MNES Inc and Bionics co., ltd. outside the submitted work.

### Author Contributions

Conception and designing of the study; Kaneda Y

Data collection; Kaneda Y

Data analysis and interpretation; Kaneda Y, Akashima S

Writing this paper; Kaneda Y

Critical revision of the paper; Tanimoto T, Takahashi K, Ozaki A

All the authors read the final draft and approved submission.
